# The Medical Inspection of School Children and the Appointment of Dr. Newman

**Published:** 1907-09-28

**Authors:** 


### The Hospital

A Journal of

The Medical Sciences and Hospital Administration.

New Series. No. 27, Vol. I. [No. 1099, Vol. XLII.] SATURDAY, SEPTEMBER 28, 1907.

### The Medical Inspection of School Children and the Appointment of Dr. Newman.

It is invidious and futile to engage in comparisons of the respective merits of appointed and disappointed aspirants to office, and we regret that an esteemed and valued contemporary should have been betrayed into so unwonted and ungracious a course when dealing with the appointment of Chief Medical Officer to the Board of Education. We do not propose to enter into the controversy of the comparative claims of Dr. Newman and Dr. Kerr to the office in question, but since the wisdom of the choice of Dr. Newman has been seriously called in question we wish again to express our emphatic approval of his appointment and our appreciation of his eminent claims to the office. It may be, as has been said, that Dr. Newman's experience of pedagogic medicine is less intimate than that of others who could be named, but as we conceive the duties of the Chief Medical Officer to the Board of Education, this is less of a disability than would from a somewhat narrow standpoint, appear.

There is no mystery in pedagogic medicine. It is, after all, only the application of common, everyday medicine to scholars and schools. The medical inspection of school children does not involve an acquaintance with a new science, the monopoly of an uncreated order of medical pedagogues. It is fortunate that no such demands are to be made on the resources of the profession in the administration of the new Act, or whence are these practitioners to be drawn? We need only ask as to the manner in which the need for the medical inspection of school children has been demonstrated, to see at once that what pre-eminently is needed to secure the successful working of the Act is a capable administrator, experienced in the working of public health law and in the ways of local government.

The Board of Education has been happy in securing in Dr. Newman an officer who has established a reputation for sound administrative ability, and one who will bring to bear a ripe executive experience which may be relied upon to secure harmony and unity in public health administration, so wisely expanded by its inclusion of the great branch of school hygiene. The plain fact is that the controversy to which we have referred is not a personal one.

The issue is the creation of a new and independent branch of local health administrators, working beside, but independent of, the existing public health service, or the expansion of the public health service so as to include the scarcely distinguishable branch of hygiene relating to scholars and schools. Dr. Newman's appointment has been interpreted as the signal that the Board of Education will view school hygiene as co-ordinate with, and administratively inseparable from, the other problems of public health. It has been offensively and most unjustifiably urged that this will mean the reduction of school hygiene to a question of "school drains." Both in the lay and a section of the medical press there has been an effrontery of assumption that the medical officer of health was concerned almost exclusively with questions of drainage, and that his interests and knowledge of preventive medicine were limited to sanitary appliances, which can only be excused on the plea that the writers were scandalously ignorant of the nature of the work which they thus travestied.

It is good that this campaign of implicit calumny has been without avail. It would be opposed to public and medical interests were there to be dual officers working independently in the medical service of the local authorities. We have repeatedly insisted on the unity of Medicine, a unity which should be preserved in proportion as it becomes increasingly related in an official capacity to the public.

The growth of the public health service, as a necessary outcome of the increased work from the medical inspection of scholars, will not be less a gain to medicine because it is less inchoate and commands a wider public and medical interest than would a brand-new order of officials medically interested in a little known fragment of pedagogics.

But the chief advantage of expanding the scope of the existing departments of public health is the efficiency with which the work of medical inspection will be carried out. Brought into relation with the other work of the public health departments, school hygiene will assume its due proportion in the public health perspective. Its importance will not be dimmed by the fact that those who would then have to deal with it are already familiar with the *rôle*

played by undetected disease and physical shortcoming among children attending public elementary schools. But just because the purview is broader and the outlook less circumscribed, will the work take its place without dislocation in the growing hegemony into which public health administration is consolidating. We hail the appointment of Dr. Newman as a sign that the central authorities are bent on maintaining an unbroken growth in the evolution of public health administration in this country.

